# 3D Bioprinting Pluripotent Stem Cell Derived Neural Tissues Using a Novel Fibrin Bioink Containing Drug Releasing Microspheres

**DOI:** 10.3389/fbioe.2020.00057

**Published:** 2020-02-11

**Authors:** Ruchi Sharma, Imke P. M. Smits, Laura De La Vega, Christopher Lee, Stephanie M. Willerth

**Affiliations:** ^1^Department of Mechanical Engineering, University of Victoria, Victoria, BC, Canada; ^2^Department of Biomedical Engineering, Eindhoven University of Technology, Eindhoven, Netherlands; ^3^Djavad Mowafaghian Centre for Brain Health, The University of British Columbia, Vancouver, BC, Canada; ^4^Division of Medical Sciences, University of Victoria, Victoria, BC, Canada

**Keywords:** tissue engineering, regenerative medicine, small molecules, drug delivery, guggulsterone, stems cells

## Abstract

3D bioprinting combines cells with a supportive bioink to fabricate multiscale, multi-cellular structures that imitate native tissues. Here, we demonstrate how our novel fibrin-based bioink formulation combined with drug releasing microspheres can serve as a tool for bioprinting tissues using human induced pluripotent stem cell (hiPSC)-derived neural progenitor cells (NPCs). Microspheres, small spherical particles that generate controlled drug release, promote hiPSC differentiation into dopaminergic neurons when used to deliver small molecules like guggulsterone. We used the microfluidics based RX1 bioprinter to generate domes with a 1 cm diameter consisting of our novel fibrin-based bioink containing guggulsterone microspheres and hiPSC-derived NPCs. The resulting tissues exhibited over 90% cellular viability 1 day post printing that then increased to 95% 7 days post printing. The bioprinted tissues expressed the early neuronal marker, TUJ1 and the early midbrain marker, Forkhead Box A2 (FOXA2) after 15 days of culture. These bioprinted neural tissues expressed TUJ1 (15 ± 1.3%), the dopamine marker, tyrosine hydroxylase (TH) (8 ± 1%) and other glial markers such as glial fibrillary acidic protein (GFAP) (15 ± 4%) and oligodendrocyte progenitor marker (O4) (4 ± 1%) after 30 days. Also, quantitative polymerase chain reaction (qPCR) analysis showed these bioprinted tissues expressed *TUJ1, NURR1* (gene expressed in midbrain dopaminergic neurons), *LMX1B*, *TH*, and *PAX6* after 30 days. In conclusion, we have demonstrated that using a microsphere-laden bioink to bioprint hiPSC-derived NPCs can promote the differentiation of neural tissue.

## Introduction

3D bioprinting has become an increasingly popular strategy for engineering tissues as shown in recent reviews (Gu et al., 2018; Tasnim et al., 2018; De La Vega et al., 2019; Salaris and Rosa, 2019). This process combines cells with bioinks, which are optimized to encourage the formation of target tissues, and deposits them into 3D structures based on specifications given in a digital design file. The properties of the bioink will also determine how well the bioprinted tissue replicates the physiology of the target tissue or organ being printed (Panwar and Tan, 2016; Gungor-Ozkerim et al., 2018; Gopinathan and Noh, 2018). Bioinks should possess a number of characteristics, including high biocompatibility, printability, and the ability to deliver factors to promote the desired behavior from the cells seeded inside. The properties of these bioinks can be tuned for printing specific tissue types as well as to support specific cell populations. In particular, hydrogels often possess the desired characteristics necessary for bioprinting tissues, in terms of viscosity and dealing with the shear stress generated during printing (Gao et al., 2019).

Human induced pluripotent stem cells (hiPSCs) were discovered in 2007 when scientists determined that overexpression of certain transcription factors could revert adult human fibroblasts back into stem cells possessing the property of pluripotency (Takahashi et al., 2007). This discovery has enabled the study of many diseases as these stem cell lines can be derived from patients suffering from different diseases and disorders. Using patient derived hiPSCs lines and differentiating them into a target tissue type is a powerful way to study diseases, including those affecting the nervous system – like Alzheimer’s and Parkinson’s (Playne and Connor, 2017; Cheng et al., 2018, 2019; Penney et al., 2019). Often, research on hiPSC models of these diseases is conducted in 2D, despite brain tissue possessing a complex 3D structure. Recent work has examined the necessary conditions for 3D printing neural tissues derived from stem cells using hydrogel-based bioinks. For example, Lozano et al. (2015) successfully bioprinted brain-like structures utilizing a bioink composed of gellan gum modified with the RGD peptide containing primary cortical neurons. More recent work from the McAlpine group demonstrated that multiple neural cell types, including neural stem cells derived from hiPSCs, could be printed with relatively high levels of viability into structures that resemble the spinal cord (Joung et al., 2018). However, most of these bioprinting studies have not attempted to generate structures that resemble the brain.

Neural tissues can be generated using many different types of bioprinting technology, including extrusion-based methods, laser assisted printing, inkjet printing, and drop on demand method (Lee et al., 2018). Our lab uses the Aspect Biosystems RX1 printer with its novel microfluidic Lab-on-a-Printer technology due to its ability to protect the cells within the bioink from shear stress during printing – enabling us to maximize cell viability (Beyer et al., 2016; Bsoul et al., 2016). Our own group developed a novel fibrin-based bioink for printing hiPSC-derived neural aggregates that both maintained their viability and differentiated into mature neural tissues after 46 days of culture (Abelseth et al., 2018). This same formulation was also used to print dissociated hiPSC-derived neural progenitor cells (NPCs) that could be matured into spinal cord-resembling tissues upon treatment with specific small molecules (De La Vega et al., 2018a). This bioink formulation supported the generation of ring shaped constructs containing the human glioblastoma cell line where the tissues exhibited high levels of viability and expressed cancer associated protein markers (Lee et al., 2019). We also showed that effects of a potential glioblastoma cancer treatment were different in our 3D bioprinted model compared to 2D culture, illustrating the need for such bioprinted models of neural diseases.

One of the challenges when working with hiPSCs is ensuring their differentiation into the desired, mature phenotypes. Neural differentiation of hiPSCs can take months and require a significant amount of labor and resources (Riemens et al., 2018). One promising strategy for promoting such differentiation requires treating these hiPSCs with small molecule morphogens (Zhang et al., 2012). Our lab has extensively explored the use of small molecule morphogens encapsulated in microspheres, which degrade over time to slowly release the drug in a controlled manner, as a means to direct neural differentiation in an autonomous fashion (Gomez et al., 2015; Agbay et al., 2018; De La Vega et al., 2018b). Guggulsterone, an anti-cancer drug, is a potent agent for differentiating both human embryonic stem cells and hiPSCs into dopaminergic neurons, the cellular population affected by Parkinson’s disease (Gonzalez et al., 2013; Robinson et al., 2015). Our recent study demonstrated that we could deliver guggulsterone in a controlled fashion from microspheres as a way to engineer mature neural tissues from hiPSCs (Agbay et al., 2018).

This work now incorporates these novel guggulsterone-releasing microspheres into our fibrin-based bioink as a tool for 3D bioprinting tissues similar to that found in the brain. The goal was to generate neural tissues containing dopaminergic neurons from hiPSCs derived NPCs to model healthy brain tissue in a dish as well as to validate the bioactive properties of our microsphere-containing bioink. In this study, we bioprinted dome shaped constructs containing hiPSC-derived NPCs encapsulated inside of our bioink containing guggulsterone microspheres and characterized their properties. We printed dome shaped structures that were 1 cm in diameter in a layer-by-layer fashion, consisting of six layers. We printed two additional sets of control tissues – (1) tissues containing NPCs and treated with guggulsterone in the media, and (2) tissues containing unloaded microspheres and NPCs. All three sets of constructs were analyzed for cell viability and their expression of markers associated with neural differentiation, in particular dopaminergic neurons.

## Materials and Methods

### Expanding Neural Progenitor Cells From hiPSCs for Bioprinting

Experiments using hiPSC-derived NPCs were conducted with the approval of the University of Victoria’s Human Ethics Committee – Protocol No. 12-187. NPCs were derived from undifferentiated hiPSCs (1-DL-01 line – male, WiCell Research Institute) as described previously (Robinson et al., 2015). NPCs were cultured in STEMdiff^TM^ Neural Progenitor Medium (NPM), (STEMCELL^TM^ Technologies, Vancouver, BC, Canada), on cell culture plates coated with poly-L-ornithine (PLO, Sigma, St. Louis, MO, United States) and laminin (Sigma, St. Louis, MO, United States). The NPCs were cultured under standard conditions consisting of 5% CO_2_ at 37°C with daily media changes. Cells were cryopreserved in STEMdiff^TM^ Neural Progenitor Freezing Medium (STEMCELL^TM^ Technologies Vancouver, BC, Canada) liquid nitrogen upon reaching 80% confluence.

### Preparation of Unloaded and Guggulsterone Microspheres

Microspheres were prepared using an oil-in-water (o/w) emulsion process as previously described (Agbay et al., 2018). 2% poly (vinyl alcohol) (PVA) (Mw ∼ 13,000–23,000, 87%–89% hydrolyzed) (Sigma-Aldrich, St. Louis, MO, United States) solution was prepared by diluting PVA in de-ionized water for an hour at 85°C with 850 rpm on a magnetic mixer (Corning Life Sciences, Tewksbury, MA 01876, United States) for the water phase. Subsequently, 100 ml of 0.3% (w/v) PVA solution was prepared by dissolving 2% PVA with de-ionized water and kept at 35°C. 500 mg of poly- ε-caprolactone (PCL) (Mn ∼ 45,000), was dissolved in 3 ml of dichloromethane (DCM, Fisher Scientific, Ottawa, ON, Canada) on a magnetic mixer for 15 min at 900 rpm for making the oil phase. Later, 0.3 mg of guggulsterone (Sigma-Aldrich, St. Louis, MO, United States) was dissolved in 100% ethanol then added to the oil phase to make microspheres at a concentration of 0.6 μg/mg (w/w, guggulsterone/PCL) microspheres. Unloaded microspheres were prepared by adding an equal volume of ethanol without the drug to the oil phase. 3 ml of 2% PVA was slowly added to the oil solution to prevent disruption of the boundary layer after removal from the magnetic mixer. Afterward, an emulsion (w/o) was then achieved by vortex mixing (Fisher Scientific) at 3000 rpm for 15 s. This (w/o) emulsion was mixed into the 0.5% PVA water phase and held at 35°C at a mixing speed of 500 rpm for 4 h to evaporate of the organic solvent. Then after mixing, the microspheres were isolated by centrifugation at 4,000 rpm (Eppendorf 5810 R model with swinging bucket rotors) and washed with deionized water. The microspheres were lyophilized for 24 h and stored at −20°C. The microspheres were sterilized by low power air-plasma treatment (Harrick Plasma, Ithaca, NY, United States) for 30 s before being added to our bioink.

### Bioprinting of Neural Tissues Consisting of hiPSC-Derived NPCs and Microspheres

**FIGURE 1:**
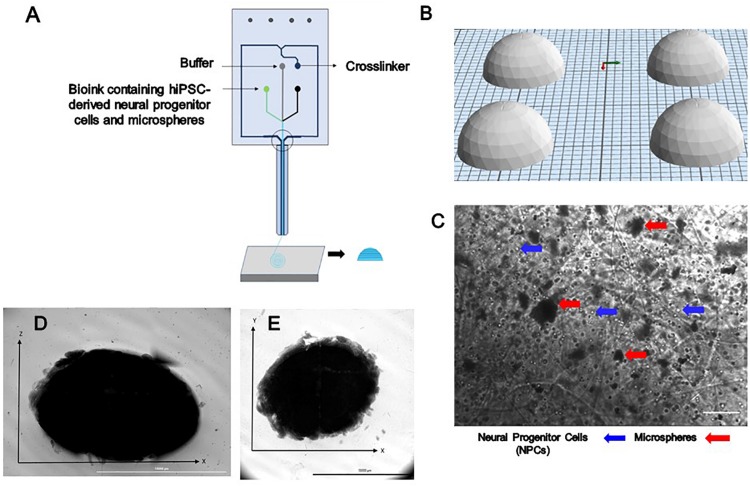
The design and printing of a dome-shaped 3D neural tissue structure. **(A)** Schematic representation of Aspect Biosystems’ microfluidic printhead. **(B)** The Computer Aided Design (CAD) file representing dome structures. **(C)** Phase contrast images of day 0 printed construct showing NPCs and microspheres are dispersed throughout the fibers within the constructs (100 μm). Top-down light microscopy image of bioprinted dome shaped construct consisting of neural progenitor cells (NPCs) with bioink containing encapsulated guggulsterone microspheres. **(D)** Image showing the side view of a bioprinted dome and **(E)** showing bottom view of the construct Scale bar for **(D,E)** represents 10,000 μm.

Bioink was prepared prior to printing as previously described (Abelseth et al., 2018). NPCs at a concentration of 1 million cells/mL were thawed and resuspended in the bioink composed of 20 mg/mL of fibrinogen (Sigma, St. Louis, MO, United States), 0.5% w/v of alginate (120,000–190,000 g/mol, M/G ratio 1.56) (Sigma, St. Louis, MO, United States), and 0.3 mg/mL of genipin (Sigma, St. Louis, MO, United States) dissolved in dimethyl sulfoxide (DMSO) (Sigma, St. Louis, MO, United States), along with 0.5 mg of microspheres in tris-buffered saline (TBS) with phenol red (Sigma, St. Louis, MO, United States) when appropriate. A 15 mL conical tube containing NPCs, bioink and when appropriate microspheres was connected to the “Material 1” channel of the Lab-On-The-Printer (LOP^TM^) printhead (Aspect Biosystems, Vancouver, BC, Canada) shown in Figure 1A. The crosslinker was comprised of 20 mg/mL of calcium chloride (Sigma, St. Louis, MO, United States), 0.075% w/v of chitosan (Sigma, St. Louis, MO, United States), and 1.7 U/mL of thrombin (Sigma, St. Louis, MO, United States) in a conical connected to the crosslinker channel. Cross-linking occurs at the junction of the bioink and crosslinker channels in the printhead (Figure 1A). Genipin was included in the bioink solution to avoid cross-linking of the chitosan present in the cross-linker solution before printing. Dome shaped constructs shown in Figures 1D,E were bioprinted based on the specifications detailed in the relevant CAD file (Figure 1B) generated using Aspect’s studio software (V1.2.59.0, Aspect Biosystems, Vancouver, BC, Canada) using a rectilinear infill pattern in a repeated layer by layer fashion. The resulting constructs consisted of 6 deposited layers of cell laden bioink. Specific pressures are applied to each channel to monitor the flow rate to provide sufficient time for the crosslinking reaction to occur. The printing speed used was 25 mm/s and pressure for bioink, crosslinker and buffer channels were 50 mbar, 60 mbar, and 100 mbar, respectively. The bioprinted groups included constructs containing guggulsterone microspheres constructs containing unloaded microspheres, and control constructs soluble guggulsterone (SG). The bioprinted constructs were transferred to 12 well cell culture plates (Greiner Bio-One GmbH, Kremsmünster, Austria) coated with PLO and laminin and incubated at 37°C in 5% CO_2_.

### Culture of Bioprinted Constructs

The bioprinted constructs were initially cultured in STEMdiff^TM^ Neural Progenitor Media (NPM) (STEMCELL^TM^ Technologies, Vancouver, BC, Canada), on cell culture plates coated with poly-L-ornithine (PLO, Sigma, St. Louis, MO, United States), and laminin (Sigma, St. Louis, MO, United States). with 1% Antibiotic Antimitotic Solution (AAS) (Sigma-Aldrich, St. Louis, Missouri, United States) for the first 10 days after printing. This media contains both epidermal growth factor and basic fibroblast growth factor to promote proliferation of hiPSC-derived NPCs. On day 10, the NPM was replaced by STEMdiff^TM^ Neural Induction Medium (NIM) (STEMCELL^TM^ Technologies, Vancouver, BC, Canada) with 1% AAS to promote maturation of the hiPSC-derived NPCs toward mature neurons as it contains the small molecules SB431542, LDN193189, and rock inhibitor Y-27632. On day 20, the NIM was replaced by Brain Phys Neuronal Medium (STEMCELL^TM^ Technologies, Vancouver, BC, Canada) for all groups to promote further maturation of these bioprinted tissues. The media changes were performed after every 2 days by replacing 50% of media for the first 30 days of culture. Phase contrast imaging was performed with a Leica DMI3000B (Leica Biosystems, Wetzlar, Germany) microscope a QImaging RETIGA 2000R camera (QImaging, Surrey, BC, Canada) at 10X magnification. Imaging of whole bioprinted construct was performed using the Cytation 5^TM^ Gen5 imager and its associated software version 3.05 (BioTek instruments, Winooski, VT, United States).

### Assessment of Cell Viability Post Printing

The bioprinted constructs were degraded using the Neural Tissue Dissociation Kit- Postnatal Neurons (Miltenyi Biotec GmbH, Bergisch Gladbach, Germany) in combination with gentleMACS^TM^ Dissociator (Miltenyi, Biotec GmbH, Bergisch Gladbach, Germany) on day 1 and 7 to obtain single cell suspensions for analysis. This process utilizes an optimized combination of enzymatic and mechanical degradation to obtain single cell suspensions. The bioprinted constructs were transferred from each group in to gentleMACS C-tube (Miltenyi Biotec GmbH, Bergisch Gladbach, Germany), from 12 well plate and later, wells of plate were washed with 1960 μL of Enzyme Mix 1 and then that enzyme added into the gentleMACS C-tube. Later, a tightly closed C tube was attached upside down on to the sleeve of gentleMACS Dissociator. Subsequently, the optimized, pre-set gentleMACS program m_brain_01 was run twice on Dissociator for 30 sec each and then constructs incubated for 20 min at 37°C. 45 μL of Enzyme Mix 2 was added to C-tubes and the pre-set gentleMACS program m_brain_02 was run twice for 30 sec each and incubated for 20 min at 37°C then finally, pre-set gentleMACS program m_brain_03 was run twice for 1 min each. Lastly, 2 mL of Fetal Bovine Serum (FBS) (Gibco^TM^, Thermo Fisher Scientific, Waltham, MA, United States) was added to the mixture to quench the enzymatic reaction and then the cell suspension was run through a 37 μm strainer (STEMCELL^TM^ Technologies, Vancouver, BC, Canada) and centrifuged at 300 × *g* to pellet the cells. The supernatant was removed, and the pellet was resuspended in 1 mL of phosphate buffered solution (PBS) (Thermo Fisher, Waltham, MA, United States). 20 μL of the cell suspension was mixed with 380 μL of Guava ViaCount reagent^®^ (Millipore, Burlington, MA, United States). 100 μL of this mixture was added to the individual wells of the 96-well plate and cell viability was determined using the Guava EasyCyte HT flow cytometer (Millipore, Burlington, MA, United States).

### Characterization of Bioprinted Constructs by Immunocytochemistry

Immunofluorescent staining was performed to assess the cell markers expressed by the bioprinted constructs on day 15 and 30. The constructs were fixed with 10% formalin at 4°C for 2 h then permeabilized with 0.1% Triton X (Sigma, St. Louis, MO, United States) at 4°C for 45 min and blocked with 5% Normal Goat Serum (Sigma, St. Louis, MO, United States) and incubated at 4°C for 2 h at 2 rpm on the shaker (The Belly Dancer^®^ orbital shaker) (Sigma-Aldrich Canada Co., Oakville, ON, Canada). The constructs then were incubated with the primary antibody FOXA2 (1:400, AbCam, Eugene, OR, United States) and anti-β-tubulin III (TUJ1) (1:400, Sigma-Aldrich Canada Co., Oakville, ON, Canada) after 15 days of culture. For day 30 constructs, the primary antibodies used were tyrosine hydroxylase (TH) (1:400, Pelfreeze, Arkansas, United States) and TUJ1 (1:400, Sigma-Aldrich Canada Co., Oakville, ON, Canada). The constructs were incubated at 4°C overnight at 100 rpm following three washes with PBS for 15 min at 4°C. Secondary antibodies Alexa Fluor 568 Donkey Anti-Mouse (1:500, AbCam, Eugene, OR, United States), and Alexa Fluor 488 Donkey Anti-Rabbit (1:400, Abam, Eugene, OR, United States) diluted in PBS were added to the constructs. Later, those incubated for an 1 h at room temperature and 3 h at 4°C on the shaker. After incubation with the secondary antibody, cells were washed in PBS three times for 15 min at 2 rpm on the shaker. The cells were counterstained with DAPI (4′,6-diamidino-2-phenylindole) nucleic acid stain (Thermo Fisher Scientific, Waltham, MA, United States). 300 μL of 300-nM DAPI solution in PBS was added to the cultures after the final wash and incubated for 3 min, followed by rinsing with PBS. The bioprinted constructs were then visualized with FIPS – Zeiss Confocal Laser Scanning Microscope (“Objective: 0,” Immersion = “Air,” Model = “EC Plan-Neofluar 20 × /0.30 M27”; Carl Zeiss Microscopy GmbH, Jena, Germany). The excitation and emission wavelengths used for detecting Alexa Fluor 488 were 479 nm and 519 nm and for detecting Alexa Fluor 588 were 580 nm and 602 nm. The pixel size for 10 × were 1040 × 1040 and 20 × was 710 × 532. The interval used is 10 microns with 20–30 slices in the z stack.

### Characterization of Bioprinted Constructs by Flow Cytometry

Bioprinted constructs were analyzed at day 30 using flow cytometry for the following markers: β-tubulin III (βT-III) (TUJ1) (R&D systems, Minneapolis, MN, United States). O4 (oligodendrocytes progenitor marker) (R&D systems, Minneapolis, MN, United States), Anti-Tyrosine Hydroxylase (TH) antibody (AbCam, Eugene, OR, United States), and GFAP (glial fibrillary acidic protein) (AbCam, Eugene, OR, United States) (a mature marker for astrocytes). The bioprinted constructs were degraded and the resulting cell suspension was processed as previously reported in see section “Assessment of Cell Viability Post Printing.” Briefly, the cell suspension was washed three times with PBS by centrifuging at 300 *g* for 5 min. The cell suspension was then fixed and stained per the manufacturer’s instructions (R&D Systems, Minneapolis, MN, United States). Isotype controls consisted of mouse IgG2A PerCP-conjugated Isotype control (R&D systems, Minneapolis, MN, United States), normal mouse IgM PE-conjugated Control (R&D systems, Minneapolis, MN, United States) and mouse IgG2b, kappa monoclonal [7e10g10] – Isotype control (AbCam, Eugene, OR, United States). The analysis was performed using the Guava EasyCyte HT flow cytometer (Millipore, Burlington, MA, United States).

### Characterization of Bioprinted Constructs by Quantitative Reverse Transcriptase Polymerase Chain Reaction (qPCR) Analysis

Total RNA was isolated from the bioprinted constructs using an RNeasy Plus Mini kit according to manufacturer’s instructions (Qiagen, Hilden, Germany). RNA content and quality as indicated by the A260/A280 ratio was measured using a NanoVue Plus (GE Healthcare, Chicago, IL, United States). Only samples with an A260/A280 ratio over 1.8 were used. One step PCR was performed on the isolated RNA as per manufacturer’s instructions for the QuantiTect SYBR Green Master Mix (204243, Qiagen, Hilden, Germany). RNA isolated from the bioprinted tissues was added to the individual wells of 96 well plates containing reaction mix. This procedure included a reverse transcriptase step followed by the PCR reaction. PCR reactions were performed in triplicates using the relevant QuantiTect Primer Assay or primers in combination with QuantiTect SYBR Green master mix to determine the levels of gene expression. mRNA levels were quantified using the primers for the following genes: Glyceraldehyde-3-phosphate dehydrogenase (*GAPDH* – served as our housekeeping gene, Eurofins Genomics, Luxembourg City, Luxembourg). β-tubulin III (*Tubb3* – plays important roles in axon guidance and maintenance, Qiagen, Hilden, Germany), tyrosine hydroxylase (*TH* – encodes the enzyme tyrosine hydroxylase, Qiagen, Hilden, Germany), nuclear receptor subfamily 4, group A, member 2 (*Nr4a2* also known as *Nurr1*, – plays a role in the differentiation and maintenance of meso-diencephalic dopaminergic neurons, Qiagen, Hilden, Germany), Paired box protein 6 (*PAX6* – promotes neural stem cell proliferation, Qiagen, Hilden, Germany), *LMX1B* (Qiagen, Hilden, Germany) using an Applied Biosystems StepOnePlus Real-Time PCR System (Foster City, CA, United States). Information on the primer assays used can be founded in Supplementary Table S1.

### Statistical Analysis

Results are presented as the mean values ± standard deviation. Statistical analysis was performed on viability, flow cytometry and qPCR using the one-way ANOVA followed by Tukey’s *post hoc* analysis using GraphPad prism 5 statistics software with *p* < 0.05 (95% confidence level) indicating minimal significance.

## Results

### Generation of Bioprinted Constructs Containing NPCs and Microspheres

**FIGURE 2:**
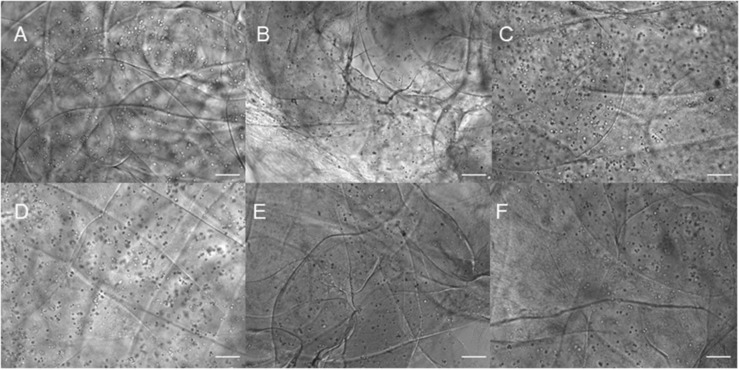
Phase contrast imaging of bioprinted constructs treated with soluble guggulsterone (SG) at **(A)** Day One and **(D)** Day 7, bioprinted constructs containing unloaded microspheres (UM) at **(B)** Day One and **(E)** Day Seven, and bioprinted constructs containing guggulsterone microspheres (GM) at **(C)** Day One and **(F)** Day Seven. Scale bars represent 50 μm.

Three different types of dome shaped bioprinted constructs containing healthy hiPSC-derived NPCs were printed from the corresponding computer aided design (CAD) file: NPCs only treated with guggulsterone in the media as a positive control referred to as SG, NPCs along with blank microspheres as a negative control referred to as UM, and NPCs along with guggulsterone releasing microspheres referred to as GM. The constructs showed a homogenous distribution of both NPCs and microspheres after printing (Figure 1C). Phase microscopy of the whole constructs showed maintenance of the dome shape post-printing. The structure comprised of 1 cm diameter – dome shape with six layers of fibers with an average width of ∼1.1 cm and height ∼ 0.7 cm (Figures 1D,E). Further phase microscopy imaging of the construct on day 1 revealed that the cells and microspheres were spread consistently throughout the construct for all culture conditions (Figure 2). While these images only represent the dispersity throughout the whole construct in the *x*-*y*-direction, but we observed similar distributions at various layers using phase microscopy, suggesting an even distribution throughout the construct.

### Cell Viability Analysis of the Bioprinted Tissues

**FIGURE 3:**
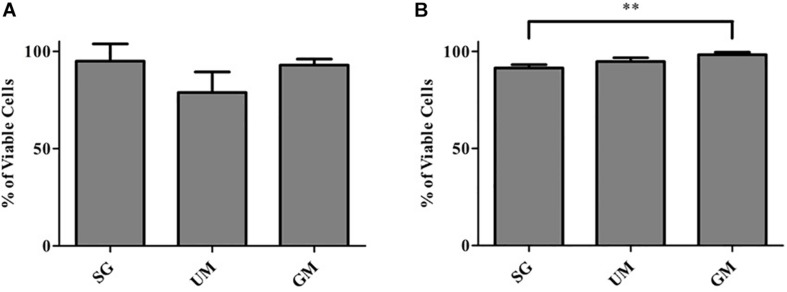
Cell viability analysis for all groups, including the bioprinted constructs treated with soluble guggulsterone (SG), constructs containing unloaded microspheres (UM) and constructs containing guggulsterone microspheres (GM) determined at **(A)** Day 1 and **(B)** Day 7 after being bioprinted. Data is reported as the mean ± S.D (*n* = 3. ^∗∗^*p* < 0.01 by one-way ANOVA and Tukey *post hoc* test for significance between samples).

Cell viability of post-printed NPCs was quantified after days 1 and 7 of culture *in vitro* (Figure 3). Constructs from all groups showed high viability 1-day post-printing: GM (92 ± 3%), UM (78 ± 11%), SG (89 ± 2%), with no statistical significance between groups observed. The GM group exhibited the highest level of viability on day 7 (98 ± 1%) in comparison with the other two groups (UM – 94 ± 2% and SG – 91 ± 2%). Overall, all groups exhibited high levels of viability post printing. The data is reported as the mean ± S.D (^∗∗^*p* < 0.01 by one-way ANOVA and Tukey *post hoc* test for significance between samples).

### Immunocytochemistry Analysis of the Bioprinted Tissues

**FIGURE 4:**
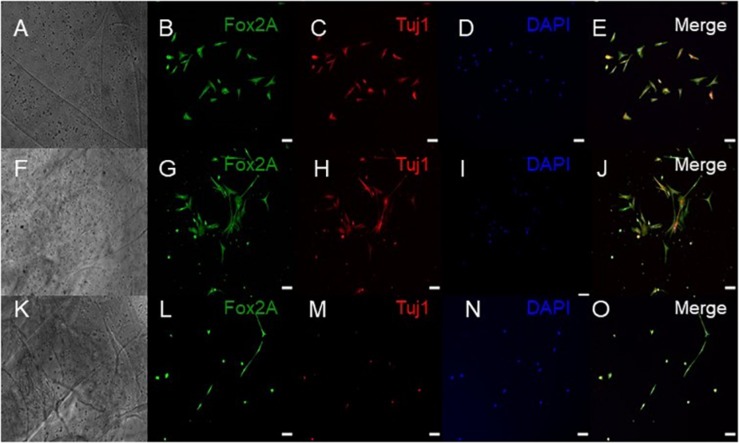
Immunocytochemistry was performed after 15 days of culture for the following markers: FoxA2 (a marker expressed by midbrain-type dopamine neurons shown in green), TUJ1 (an early marker for neurons shown in red), and the nuclear stain DAPI, (4’,6-diamidino-2-phenylindole shown in blue). **(A–E)** shows bioprinted tissues treated with soluble guggulsterone (SG), **(F–J)** shows bioprinted tissues containing unloaded microspheres (UM), and **(K–O)** shows bioprinted tissues containing guggulsterone microspheres (GM). The scale bar is 100 μm.

**FIGURE 5:**
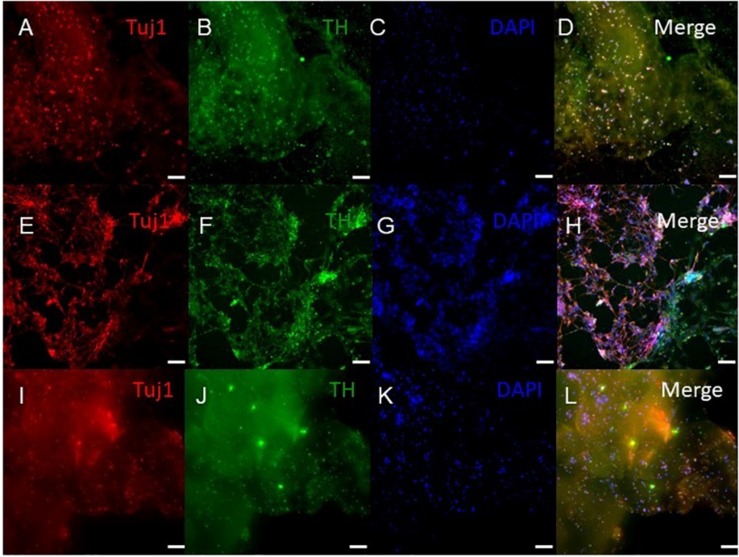
Immunocytochemistry was performed after 30 days of culture on cell that migrated out of the bioprinted constructs for the following markers: TUJ1 (an early marker for neurons shown in red), TH (a dopaminergic neuron marker shown in green), and the nuclear stain DAPI shown in blue. **(A–D)** shows bioprinted tissues treated with soluble guggulsterone (SG), **(E–H)** shows bioprinted tissues containing unloaded microspheres (UM), and **(I–L)** shows bioprinted tissues containing guggulsterone microspheres (GM). The scale bar is 100 μm.

**FIGURE 6:**
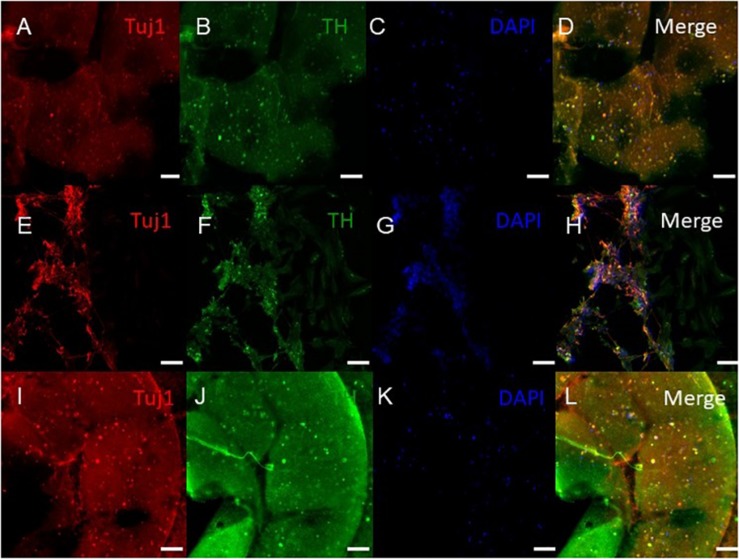
Immunocytochemistry was performed after 30 days of culture on the cells embedded in different layers of bioprinted constructs for the following markers: TUJ1 (an early marker for neurons shown in red), TH (a dopaminergic neuron marker shown in green), and the nuclear stain DAPI shown in blue. **(A–D)** shows bioprinted tissues treated with soluble guggulsterone (SG), **(E–H)** shows bioprinted tissues containing unloaded microspheres (UM), and **(I–L)** shows bioprinted tissues containing guggulsterone microspheres (GM). The scale bar is 100 μm.

ICC was performed on constructs for all three groups for the cellular markers TUJ1 (an immature neuronal maker) and FOXA2 (a midbrain-type dopamine neuron marker) at day 15 (Figure 4) and on day 30 for TUJ1 and TH (an enzyme expressed by dopaminergic neurons) (Figures 5, 6). All constructs stained positive for varying levels of TUJ1 and FOXA2 on day 15. Similarly, all constructs expressed TUJ1 on day 30 with the GM and SG tissues expressing TH as well.

### Flow Cytometry Analysis of the Bioprinted Tissues

**FIGURE 7:**
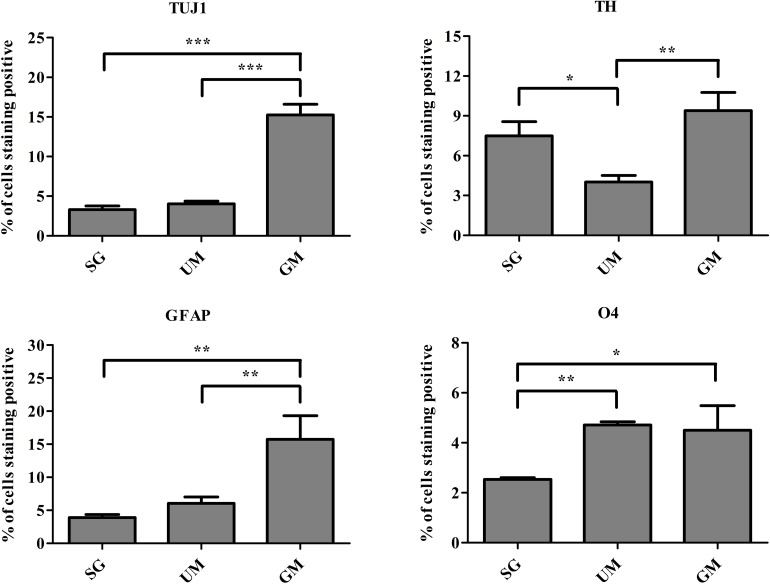
Quantitative flow cytometry assessment of the cell types present on day 30 in the bioprinted constructs treated with soluble guggulsterone (SG), bioprinted constructs containing unloaded microspheres (UM), and bioprinted constructs containing guggulsterone microspheres (GM) for the following markers: TUJ1 (an early marker for neurons), tyrosine hydroxylase (TH, a dopaminergic neuronal marker), glial fibrillary acidic protein (GFAP, a protein expressed by astrocytes), O4 (a marker expressed by oligodendrocytes). Data is reported as the mean ± SD (*n* = 3; *p < 0.05, **p < 0.01, ***p < 0.001 by one-way ANOVA and Tukey *post hoc* test for significance between samples for all groups.

Flow cytometry was performed to quantify the percentage of cells expressing the following markers: TUJ1, TH, glial fibrillary acidic protein (GFAP, marker expressed by astrocytes), and O4 (an oligodendrocyte marker) on day 30 (Figure 7). Expression of TUJ1 was significantly higher for the GM tissues (15 ± 1%), followed UM (4 ± 1%), SG (3 ± 1%). Accordingly, expression of TH was the highest for the GM tissues (8 ± 1%), followed by the SG group (7 ± 1.0%) and then UM group has the lowest expression level (4 ± 1%). GFAP expression was the highest for the GM group (15 ± 4%) followed by the UM group (6 ± 1%), with the SG group having the lowest expression levels (3 ± 1%). Finally, both the GM and UM groups had similar levels of O4 expression (5 ± 1%) compared to SG (3 ± 1%). Overall, the guggulsterone microspheres promoted more mature differentiation of the bioprinted NPCs seeded inside of our engineered tissues. The data is reported as the mean ± SD (*n* = 3; *p < 0.05, **p < 0.01, ***p < 0.001 by one-way ANOVA and Tukey *post hoc* test for significance between samples for all groups.

### QPCR Analysis of the Bioprinted Tissues

**FIGURE 8:**
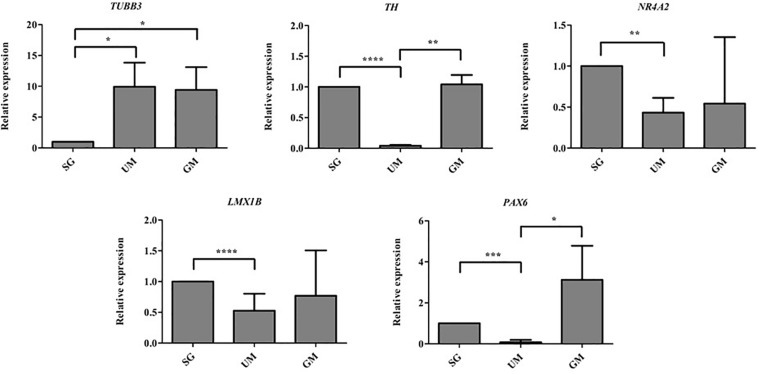
qPCR was performed on Day 30 to determine the relative gene expression levels of the following neurodevelopmental genes: β-tubulin III (*Tubb3*), tyrosine hydroxylase (*TH*), nuclear receptor subfamily 4, group A, member 2 (*Nr4a2* also known as *Nurr1*), LIM homeobox transcription factor 1β (*LMX1B*), Paired box protein 6 (*PAX6*). Gene expression levels were normalized to *GAPDH* and then to levels expressed the bioprinted constructs treated with soluble guggulsterone (SG). Data is reported as the mean ± SD (*n* = 3; *p < 0.05, **p < 0.01, ***p < 0.001, ****p < 0.0001 by one-way ANOVA and Tukey *post hoc* test for significance between samples).

Quantitative polymerase chain reaction was performed to analyze the gene expression levels present in our three different groups of bioprinted tissues on day 30 and the gene expression levels were normalized to the positive control – soluble guggulsterone in the media (Figure 8). Both sets of tissues containing microspheres showed increased levels of *TUBB3* (gene encoding for *TUJ1*) in comparison to the tissues treated with soluble guggulsterone as well reduced levels of *NR4A2* (Nurr1) (dopaminergic neurotransmitter phenotype gene). Interestingly, both the SG and GM groups showed higher levels of TH RNA in comparison to the UM group. The tissues showed decreased *LMX1B* expression in comparison to the tissues treated with soluble guggulsterone. Finally, the GM group also exhibited the highest levels of *PAX6* RNA (a neural progenitor marker).

## Discussion

3D bioprinting combines cells with biocompatible materials to create 3D structures with defined micro and macro architectures (Hsieh and Hsu, 2015). In comparison to traditional 2D cultures, 3D bioprinted tissues provide an improved platform for mimicking tissues *in vitro*. In particular, 3D structures can replicate the influence of the microenvironment on cell growth as well as cell-cell and cell-matrix interactions (Lee et al., 2010). Such bioprinted microenvironments can promote the differentiation of hiPSC-derived NPCs into mature, electro-physiologically active neurons. Our group has engineered 3D bioprinted hiPSC-derived neural tissue that mimics spinal cord tissue by treating these tissues with a variety of small molecules (De La Vega et al., 2018a). While these 3D bioprinted constructs show promise as an *in vitro* neural tissue models, there is still significant room for improvement. Traditional neural differentiation methods require supplementing media with small molecules and growth factors is the conventional technique for inducing neural differentiation. Incorporating drug releasing microspheres in our bioinks can improve the differentiation efficiency of the cells inside while minimizing the number of media changes. As such, our 3D bioprinted constructs could be improved by increasing distribution of differentiation factors within the bioinks. 3D bioprinted constructs containing microspheres and compared their properties to pure bioprinted hydrogels and found incorporation of microspheres enhanced cell viability in the 3D constructs (Tan et al., 2016). The goal of this study was to improve functional maturation of 3D printed neural tissue models by incorporating drug releasing microspheres in our bioink. In particular, the incorporation of guggulsterone releasing microspheres in our bioink was evaluated as a method to induce cells to differentiate toward a dopaminergic neuronal fate. 3D printing enables the generation of objects with geometric structures that would be difficult to produce using traditional tissues engineering methods. In the present study, we have focused on bioprinting of dome-shaped constructs containing NPCs to produce a functional tissue with a homogeneous distribution of cells throughout the construct so they can interact in three dimensions. Additionally, this shape more accurately replicates the microenvironment in the brain compared to cross-hatched structures printed in previous studies (Gu et al., 2016). Finally, our dome-shaped constructs possessed a porous structure that enabled transfer of nutrients and oxygen, allowing the long-term culture of cells *in vitro*.

First, we successfully bioprinted NPCs in combination with drug releasing microspheres containing guggulsterone to create a complex tissue model the using Aspect Biosystems RX1 bioprinter. Phase contrast microscopy revealed that post printing cells are homogenously placed with microspheres throughout the fibers in different layers (Figure 1C). Most of the researchers focused on bioprinting neural stem cells (NSCs) with different biocompatible materials and differentiating them with several factors in to mature neurons and glial cells (Hsieh and Hsu, 2015; Gu et al., 2016; Huang et al., 2017; Zhou et al., 2018). While previous research using our bioink showed high cell viability post printing (De La Vega et al., 2018a; Lee et al., 2019), the effect of the addition of microspheres had not been studied. Here we investigated bioprinting NPCs along with guggulsterone releasing microspheres for generating tissues containing mature neurons. The bioprinted tissues containing NPCs showed high levels of viability on both day 1- and 7-day post printing. Cell viability for GM and SG was 92% and 94%, respectively, 1 day post printing while the UM group had 78% cell viability. These percentages are higher than those reported by Gu et al. (2016) where immediately after printing using a bioink made up of alginate, carboxymethyl chitosan and agarose 25% of frontal cortical human NSCs died. Additionally, Joung et al. (2018) reported the cell viability of spinal NPCs printed in hydrogel matrices consisting of gelatin methacrylate (GelMa) and gelatin mixed with fibrin ranged from 75 to 88% after 3 h and later decreased to 50% after 1 day. Later, bioprinted iPSC-derived spinal NPCs and oligodendrocyte progenitor cells (OPCs) after 4 days remained 75% viable in a 50% Matrigel bioink. Thus, our bioprinting process preserves cell viability at higher levels than previously reported by other groups.

Cell viability was above 90% for all groups, where GM and UM showed the highest levels of cell viability at 98% and 94%, respectively, and SG showed 91% cell viability on day 7. These percentages are higher than those reported by Salaris et al. (2019) where cell viability of NPCs were 71% after 7 days of culture in a bioink comprised of a Matrigel/alginate solution (Salaris et al., 2019). De La Vega et al. (2018a) reported the cell viability of hiPSC-derived NPCs as >81%. Additionally, Tan et al. (2016) reported the post printing viability of bioprinted mouse fibroblasts L929 cells with poly (D,L-lactic-co-glycolic acid) (PLGA) microspheres was greater than 90% after 2, 7, and 14 days. They reported that microspheres provide a cushion around the cells for preventing shear stress produced during printing process and post printing. This study has demonstrated that these bioprinted constructs containing microspheres provide a suitable 3D environment for different types of cells to grow. Importantly, our work here corroborates that the addition of microspheres does not negatively affect cell viability within the printed constructs. Furthermore, the increased viability on day 7 suggests that cells adapted to the scaffold microenvironment in the presence of microspheres, which enabled the cells to positively proliferate. The microspheres also became less prominent over time, suggesting that they were being degraded by the presence of cells.

Our 3D bioprinted tissues were cultured for 15 and 30 days *in vitro* for analysis of the tissue composition which longer than done in previous studies where ICC staining was performed after 12 days post printing (Zhou et al., 2018). 3D bioprinted constructs showed positive staining for the TUJ1 and FOXA2 at day 15 and expression of TH at day 30. FOXA2 was positively expressed in SG, GM, and UM. Several studies have suggested that FOXA2 plays an important role in directing NPCs to differentiate into dopaminergic neurons and its expression is critical for phenotype maintenance, function and survival in this neuronal subtype (Stott et al., 2013; Kim et al., 2017). Its upregulation in all groups indicates that 3D bioprinted environment enabling NPCs toward the dopaminergic neuron fate (Figure 4). Zhou et al. (2018) cultured their bioprinted constructs for 12 days to assess the potential of 3D (GelMA)-functionalized dopamine (DA) scaffolds to induce neuronal differentiation and demonstrated significant TUJ1 staining was noted on GelMA and GelMA–DA scaffolds over time. In present study, at day 30, TUJ1 was positively expressed by GM and SG in similar way when compared to UM. Upregulation of TUJ1 in all of our bioprinted tissue conditions suggests cells are adopting moving further toward a neuronal fate. In comparison with UM; SG and GM expressed comparatively more of the TH enzyme that synthesizes dopamine (Zhou et al., 2018), and its upregulation in SG and GM imply the adoption of a dopaminergic fate due to the presence of guggulsterone. Interestingly, in Gu et al. (2017), their bioprinted constructs had expressed mature neuronal markers, such as microtubule associated protein 2 (MAP2), gamma-aminobutyric acid (GABA), and Synaptophysin at day 40 though we did not examine these markers in our current study.

Flow cytometry was performed to quantify observed changes in neural marker expression. Previously, Gu et al. (2016) reported low levels expression of TUJ1 (2%) after 21 days of differentiation for bioprinted hiPSC-derived NPCs. These levels of TUJ1 expression are significantly higher in our studies at day 30. The expression was observed to be higher in GM tissues when compared to UM and SG tissues, implying the delivery of guggulsterone through microspheres provided the best environment for neuronal differentiation. Interestingly, SG tissues had the lowest expression of TUJ1. It may be that soluble drug did not influence differentiation of cells embedded in the bioink to same extent as the delivery of guggulsterone by microspheres. Previous work from our group has shown how such drug releasing microspheres can promote differentiation of hiPSC-derived NPCs into mature neural tissues (Agbay et al., 2018). Additionally, the UM group expressing higher levels of TUJ1 than SG implies that the presence of the physical presence of the microspheres can influence differentiation. Previous studies also observed similar expression of TUJ1 in tissues treated with GM and SG. The same study showed that TUJ1 expression was the lowest in conditions lacking both guggulsterone and microspheres (Agbay et al., 2018). Here, we also observed that TH expression was higher in GM and SG groups than in tissues containing UM. This result was expected, as guggulsterone works as an effective inducer of pluripotent stem cell-derived neural stem cells into dopaminergic neurons (Gonzalez et al., 2013; Robinson et al., 2015). Interestingly it was observed that the percentage of cells expressing GFAP – a marker for astrocytes – was highest in the GM group. Previous studies have determined guggulsterone to be a potent inhibitor of the signal transducer and activator of transcription 3 (STAT3) pathway, an intracellular pathway responsible for directing neural progenitors toward an astroglial fate (Gonzalez et al., 2013).

The results of current studies suggest that NPCs react differently to guggulsterone when grown in a 3D environment. Consequently, this microenvironment assists NPCs to differentiate into glial fate along with TH positive neurons. Additionally, it was found that UM showed the highest percentage of cells expressing O4, suggesting that PCL microspheres preferably assisted in differentiation toward oligodendrocytes rather than neurons and astrocytes. The tissues containing GM also expressed O4, indicating that these tissues possess all three major neural subtypes – neurons, astrocytes, and oligodendrocytes.

We then used qPCR to confirm the transcriptional profile of 3D bioprinted neural tissue after 30 days. Huang et al. (2017) reported expressions of neural-related genes such as nestin, β-tubulin, and GFAP for NSCs in different hydrogel constructs after 3 days by qPCR. Moreover, Salaris et al. (2019) reported bioprinted constructs cultured for 45 days showed expression of neural progenitor markers such as *PAX6, FOXG1*, and *TBR2*, an astrocyte marker GFAP and mature cortical neuron marker TBR1. However, our study demonstrated *TUBB3 (TUJ1)* expression was observed to be higher in GM tissues alongside UM tissues when compared to SG tissues at day 30. It may be that soluble drug did not induce bioprinted NPCs to express this gene while the incorporated microspheres increased expression. A study by Gu et al. (2017) reported gene expression analysis by qRT-PCR supported by flow cytometry and immunofluorescence post printing 3 weeks showing upregulation of *TUJ1, OLIGO2*, and *GFAP*. TUJ1 was upregulated in previous studies when hiPSCs were differentiated with guggulsterone for deriving dopaminergic neurons (Gonzalez et al., 2013). Similar to the results observed using flow cytometry, the UM groups demonstrated a high expression of *TUBB3* suggests that the microspheres strongly influencing differentiation. Previous studies also confirmed PCL microspheres induced the differentiation of hiPSC-derived NPCs into neurons (Agbay et al., 2018). Thus, it is possible that the particles present in the bioink influence growth and differentiation of cells. Increased *TH* expression indicated by GM and SG group suggests that guggulsterone is an effective inducer of neural precursors into dopaminergic neurons. Previous studies indicated the importance of *NR4A2 (Nurr1) and LMX1B* in generation of dopaminergic neurons (Chinta and Andersen, 2005; Niu et al., 2018). Two transcription factors that regulate dopaminergic differentiation *NR4A2 (Nurr1)* and *LMX1B* were also more highly expressed in GM and SG groups than UM. These results indicate that guggulsterone is potentially differentiating NPCs into dopaminergic neurons. The UM group expressed lower levels of *NR4A2 (Nurr1)* and *LMX1B*, which indicates the pivotal role of guggulsterone in inducing the dopaminergic fate in these bioprinted tissues.

The transcription factor *PAX6* is known as a neurogenic determinant in adult NPCs during development, is expressed in selectively populated dopaminergic neurons, and plays a significant role in Parkinson’s disease (Sebastián-Serrano et al., 2012; Chandrasekaran et al., 2017). Higher levels of *PAX6* were observed in GM and SG when compared with the UM group. The GM and SG groups expressed *PAX6*, which implies an increased proliferation of NPCs.

*TH*, *NURR1*, and *LMX1B* mRNAs were upregulated in the guggulsterone containing group, which suggests that bioprinted NPCs possess dopaminergic fate. 3D bioprinted NPCs with GM and SG positively expressed dopaminergic neuron-enriched transcription regulators *NURR1*, *LMX1B*, FOXA2, and TH. These results were similar to other studies that used guggulsterone to derive dopaminergic neurons from hiPSCs (Gonzalez et al., 2013; Robinson et al., 2015). These results also suggest that the bioprinted tissues containing guggulsterone releasing microspheres possess gene and protein expression profiles similar to those for dopaminergic neurons.

Our results suggested that microsphere incorporated scaffolds could potentially generate dopaminergic neurons and a number of committed differentiated neurons. Once optimized, these 3D bioprinted neural tissues could be used to model neurodegenerative diseases using patient-specific hiPSC lines, as currently done in 2D (Poon et al., 2017; Fantini et al., 2019). This study provides an approach to generate 3D neural tissues containing dopaminergic neurons as a clinically relevant model for drug discovery as well as a potential way to generate tissue to replace the lost neurons that die off during Parkinson’s disease.

Our work validates that 3D-printed customizable microsphere-based bioinks can play a positive role in promoting neural differentiation into specific neuronal subtypes while maintaining high levels of cell viability. This work suggests that this technique is promising for enhancing tissue corroborated regeneration in the future. Since a major challenge in transplantation is low cell viability, our bioprinted 3D structures could provide an attractive avenue for the regeneration of cell-specific tissues. The results reported here demonstrate how the controlled release of the bioactive small molecule guggulsterone from microspheres can be used for neuronal differentiation toward dopaminergic neurons when used in combination with hiPSC-derived NPCs. Accordingly, further research could focus on increasing the efficiency of dopaminergic neurons in bioprinted neural tissues, as in previously described protocols, For example, additional microspheres delivering retinoic acid and purmorphamine along with these guggulsterone releasing microspheres could further encourage the growth and maturation of tissues (De La Vega et al., 2018b). Additionally, the controlled delivery of other signaling factors could be explored to increase the neuronal efficiency and maturation of 3D bioprinted neural tissues.

## Conclusion

Adding drug releasing microspheres to a novel bioink improves cell survival and differentiation, particularly when engineering tissue from stem cells, to indicate their value as a tool for engineering tissues. Here, we show how the controlled release of guggulsterone from microspheres can enhance the survival of NPCs present in bioprinted tissues as well as their differentiation into mature neural tissues. This work lays the groundwork for producing engineered neural tissues from pluripotent stem cells to serve as a potential tool for high-throughput drug screening.

## Data Availability Statement

The raw data supporting the conclusions of this article will be made available by the authors, without undue reservation, to any qualified researcher.

## Author Contributions

RS designed, performed, supervised the experimental procedures and data analysis, and contributed to writing, reviewing, and editing the manuscript. IS assisted on experimental set-up and data analysis. LD assisted on experimental set-up and helped with data analysis. CL helped in editing the manuscript and figures. SW provided the input into experimental design, provided the feedback and supervision on the experimental analysis, as well as writing and editing the manuscript and responding to reviewers.

## Conflict of Interest

SW has a collaborative research agreement with Aspect Biosystems to commercialize the results of their 3D printed tissues and related reagents. The remaining authors declare that the research was conducted in the absence of any commercial or financial relationships that could be construed as a potential conflict of interest.
