# Supplementary material for: 3D Bioprinting Pluripotent Stem Cell Derived Neural Tissues Using a Novel Fibrin Bioink Containing Drug Releasing Microspheres
Source: Front Bioeng Biotechnol. 2020 Feb 11;8:57. doi: 10.3389/fbioe.2020.00057 (PMC7026266; doi:10.3389/fbioe.2020.00057)
Supplement: Supplementary file 1 [file Table_1.DOCX]

**Supplementary Table One: Primer Assays used for Quantitative Polymerase Chain Reaction Analysis**

| **Assays Purchased from Qiagen** | **Information** |
| --- | --- |
| *TH* | QT00067221 QuantiTect Primer Assay |
| *PAX6* | QT00071169 QuantiTect Primer Assay |
| *NR4A2 (NURR1)* | QT00037716 QuantiTect Primer Assay |
| *TUBB3* | QT00083713 QuantiTect Primer Assay |
| *LMX1B* | QT00025746 QuantiTect Primer Assay |
|  |  |
|  |  |
|  |  |
| **Primers purchased from Eurofins** | **Sequences** |
| *GAPDH* | Forward Primer (5'-3') GGTCTCCTCTGACTTCAACA  Reverse Primer (5'-3') AGCCAATTCGTTGTCATAC |
|  |  |
|  |  |
|  |  |
